# High-dimensional linear state space models for dynamic microbial interaction networks

**DOI:** 10.1371/journal.pone.0187822

**Published:** 2017-11-15

**Authors:** Iris Chen, Yogeshwar D. Kelkar, Yu Gu, Jie Zhou, Xing Qiu, Hulin Wu

**Affiliations:** 1 Department of Biostatistics and Computational Biology, University of Rochester, Rochester, NY 14642, United States of America; 2 Department of Biology, University of Rochester, Rochester, NY 14642, United States of America; 3 Department of Statistics, Xidian University, Xian, Shanxi 71007, China; Pennsylvania State University, UNITED STATES

## Abstract

Medical researchers are increasingly interested in knowing how the complex community of micro-organisms living on human body impacts human health. Key to this is to understand how the microbes interact with each other. Time-course studies on human microbiome indicate that the composition of microbiome changes over short time periods, primarily as a consequence of synergistic and antagonistic interactions of the members of the microbiome with each other and with the environment. Knowledge of the abundance of bacteria—which are the predominant members of the human microbiome—in such time-course studies along with appropriate mathematical models will allow us to identify key dynamic interaction networks within the microbiome. However, the high-dimensional nature of these data poses significant challenges to the development of such mathematical models. We propose a high-dimensional linear State Space Model (SSM) with a new Expectation-Regularization-Maximization (ERM) algorithm to construct a dynamic Microbial Interaction Network (MIN). System noise and measurement noise can be separately specified through SSMs. In order to deal with the problem of high-dimensional parameter space in the SSMs, the proposed new ERM algorithm employs the idea of the adaptive LASSO-based variable selection method so that the sparsity property of MINs can be preserved. We performed simulation studies to evaluate the proposed ERM algorithm for variable selection. The proposed method is applied to identify the dynamic MIN from a time-course vaginal microbiome study of women. This method is amenable to future developments, which may include interactions between microbes and the environment.

## Introduction

Human epithelial surfaces such as those of intestines, mouth and vagina provide rich environment for the growth of a variety of bacteria that together constitute most of the human microbiome. It is now widely understood that microbiome has direct relationship to human health. Consequently, it is important to understand how bacteria that constitute the microbiome interact with their hosts and with each other. These bacteria interact with each other in various forms of cooperative and antagonistic relationships, and this complex set of interactions can be depicted in the form of a Microbial Interaction Network (MIN) [1].

The degree of cooperative and antagonistic relationships between two types of bacteria can be gauged from the impact that one type has over the growth and abundance of the other. Interactions among bacteria have traditionally been inferred using microbiological assays involving co-culturing; however not all bacteria can be cultured, and laboratory inferred interactions may not occur in nature. In contrast, sequencing the variable regions of 16S ribosomal RNAs directly from biological samples gives estimates of abundance of a large variety of bacteria, which can provide a holistic and unbiased view of microbial interactions.

Few longitudinal studies of human microbiome have been undertaken, and the initial discoveries include discovery of the most abundant microbial taxa on various locations on human body [2–5]; large inter-personal and within-subject temporal variation in microbiome composition [3, 6, 7]; and the effect of external stimuli on microbiome [8, 9]. However, the construction of MIN operational in human microbiota remains a major challenge due to the high-dimensional and high-fluctuation nature of the data [1].

MIN can be constructed either by cross-sectional data [10, 11] or time-series data [12–15]. Compared with MINs constructed from cross-sectional data, MINs constructed from time-course data can capture the dynamic relationship between different bacteria and/or external stimuli, which arguably provide more realistic representations of the interactions of microbiota as they operate in nature [1, 8]. In this paper, we will focus on reconstructing dynamic MINs based on time series data which also is technically more challenging compared to that based on the cross-sectional data.

Many models have been proposed for constructing dynamic MIN [10–12, 15–18], including those based on ordinary differential equation (ODE) models [13, 14]. An ODE model is formed by taking the derivative of bacterial abundance as a function of abundance of all other bacteria and/or external stimuli. This results in a directed network model, and the dynamic nature of MIN is automatically captured and quantified. However, it is computationally difficult to apply ODE models to more than a dozen or so variables to simultaneously estimate system dynamics and regulatory relationships. Furthermore, most ODE models ignore both system and measurement errors, which in many cases have critical impact on results. In this paper, we will explore the utility of state space model (SSM), which is an alternative to ODE models, to capture MIN dynamics from time-course data.

A state space model (SSM) is a special case of dynamic Bayesian networks (DBNs). For simplicity, we only consider linear SSM, also referred to as linear dynamic systems (LDS) [19–21], for dynamic MINs in this study. SSMs have been extensively applied in the field of engineering, and recently, in systems biology, for noisy measurements over time, and to discover underlying true dynamics of the system [22]. In our study, we let $y_{t}\in R^{p}$ represent a *p*-dimensional vector of microbial abundance of *p* bacterial operational taxonomic units (OTUs) observed at time *t*. Here, OTU is an operational definition of bacterial species, obtained using clustering of 16S ribosomal RNA sequences extracted from biological samples. In linear SSMs, *y*_*t*_ is assumed to be generated from a *k*-dimensional real-valued hidden state variable vector $x_{t}\in R^{k}$, and the sequence of evolving *x*_*t*_ follows a first-order Markov process, which can be written as [20]

$$\begin{array}{c} \left\{ \begin{array}{c} x_{t}=Ax_{t-1}+w_{t},\\ y_{t}=Cx_{t}+v_{t}. \end{array},\;t=1,\ldots ,T.\right. \end{array}$$ (1)

Here *A* is the *k* × *k*-dimensional state dynamic (autoregressive) matrix; *C* is the *p* × *k*-dimensional observation matrix; and *w*_*t*_ ∼ *N*(0, *Q*), *v*_*t*_ ∼ *N*(0, *R*) are independent system and measurement noises, respectively. Both *Q* and *R* are assumed to be diagonal in many practical applications. The initial state vector $x_{0}\in R^{k}$ is usually assumed to have distribution *N*(*μ*, *Σ*).

In order to capture the dynamic MIN using state space model, it is necessary to investigate the problem of parameter estimation and variable selection for high dimensional SSMs [23, 24]. For example, Rangel and colleagues [23] applied SSMs in which observations were divided into a set of input (or exogenous) variables and a set of output (or response) variables, and the dimension was determined by cross-validations; Kojima *et al* [24] proposed a vector autoregression (VAR) model for the dynamic gene network. Based on the state space representation of VAR, they investigated the problem of parameter estimation and variable selection by L1 regularization and EM algorithm. Although these publications have suggested several useful ideas about the statistical inference of high dimensional state space model, efficient algorithms for establishing MIN have not been well addressed from a computational perspective.

In this paper, we develop a practical dynamic MIN reconstruction pipeline based on SSM that not only incorporates many existing SSM parameter estimation and model selection techniques, but also is computationally efficient and applicable for “large *p*, small *n*” data such as 16S microbiome abundance data. First, we propose a novel Expectation-Regularization-Maximization (ERM) computational framework for the SSMs, and provide a feasible implementation strategy for initialization of the ERM algorithm, *i.e.*, to initialize the ERM algorithm from the R step using nonparametrically estimated state variables instead of initializing the algorithm from the E step, which is not feasible in the high-dimensional SSM case. Second, we propose the vectorization of the matrices in the SSM and use a concept of “pseudo-regression” to justify the R step for L1-regularization based on which the standard LARS algorithm with minor modifications can be carried out. Third, a new row-based algorithm is proposed in order to reduce the memory footprint, which is a major computational cost for high-dimensional data analyses. In simulation studies we demonstrate that the proposed row-based algorithm performs equally well and could handle a higher dimensional model compared to a matrix-based algorithm. Lastly, we apply the proposed method to reconstruct dynamic MIN for normal bacterial communities in human vagina. The MINs that were constructed revealed some previously known as well as some novel microbial relationships.

## Methods

### Model selection and parameter estimation of high-dimensional linear state space models

We propose a linear SSM with an Expectation-Regularization-Maximization (ERM) algorithm to efficiently construct dynamic MINs. Since time course abundance data for all bacteria in the network can be obtained using next generation sequence technology, we can set the observation matrix *C* = *I*_*p*×*p*_ (identity matrix) in Model (1). That is, the linear SSM for dynamic MIN can be written as

$$\begin{array}{c} \left\{ \begin{array}{c} x_{t}=Ax_{t-1}+w_{t},\\ y_{t}=x_{t}+v_{t}. \end{array},\;t=1,\ldots ,T.\right. \end{array}$$ (2)

Thus, in this model, the dimension of state vector equals the dimension of observation vector (*k* = *p*). Other assumptions remain the same as in Model (1). For simplicity, we also assume that both *Q* and *R* are diagonal, *i.e.*, $Q=\sigma_{Q}^{2}\times I_{p\times p}$ and $R=\sigma_{R}^{2}\times I_{p\times p}$.

The above model allows us to use the time-course microbiome data to construct a direct dynamic MIN with distinguishable system noise and measurement noise. Each element *a*_*ij*_ (denoting the *i*th-row and *j*th-column element) in *p* × *p* system matrix *A* represents a directed edge in the network which is time-invariant, and reflects the interacting effect from bacterial species *j* to bacterial species *i*. However, when *p* is very large, it is infeasible to directly estimate *A* since we may encounter the problem of estimating a high-dimensional matrix with sparse data that requires inverting high-dimensional matrices, which not only is computationally intensive, but also can be numerically unstable. In addition, microbial interaction networks are usually sparse, *i.e.*, each bacterial species may only be impacted by a limited number of other species. In other words, high-dimensional *p* × *p* matrix *A* is a sparse matrix with many elements being zero. It is advantageous to perform variable selections to determine the zero elements of *A* while we can estimate the non-zero elements at the same time.

It is known that, from the Markov property of the state space model, the joint likelihood for complete data for the SSM can be written as

$$\begin{array}{c} P\left(\theta \right)=P\left(x_{1}\right)\prod_{t=2}^{T}P\left(x_{t}|x_{t-1}\right)\prod_{t=1}^{T}P\left(y_{t}|x_{t}\right) \end{array}$$ (3)

where *θ* = (*A*, *Q*, *R*, *μ*, *Σ*). For the linear SSM (2), the joint log-likelihood of complete data can be expressed as

$$\begin{array}{c} \begin{array}{cc} \log P(\theta ) & =-\sum_{t=1}^{T}\left(\frac{1}{2}{\left[y_{t}-x_{t}\right]}^{\prime }R^{-1}\left[y_{t}-x_{t}\right]\right)-\frac{T}{2}\log\left|R\right|\\ & \;-\sum_{t=2}^{T}\left(\frac{1}{2}{\left[x_{t}-Ax_{t-1}\right]}^{\prime }Q^{-1}\left[x_{t}-Ax_{t-1}\right]\right)-\frac{T-1}{2}\log\left|Q\right|\\ & \;-\frac{1}{2}{\left[x_{1}-\mu \right]}^{\prime }\Sigma^{-1}\left[x_{1}-\mu \right]-\frac{1}{2}\log\left|\Sigma \right|-Tp\log\left(2\pi \right). \end{array} \end{array}$$ (4)

In the following subsections, we propose the Expectation-Regularization-Maximization (ERM) algorithm to simultaneously determine the zero-elements and estimate the non-zero elements of *A* based on the maximum likelihood principle.

### Expectation-Regularization-Maximization (ERM) algorithm

When the SSM parameters are known, the Kalman filter and smoother can be used to estimate the hidden states [25]. Assuming that model parameters are *given*, the Kalman filter is the optimal method to estimate the state variables at time *t* of a linear Gaussian SSM from a sequence of noisy observations {*y*_1_, …, *y*_*t*_, …, *y*_*T*_}. Shumway and Stoffer [26] introduced an EM algorithm to estimate unknown parameters for the linear dynamic systems when the observation matrix *C* is known, such as in Model (1). The EM algorithm has gradually become a standard estimation tool for SSMs and related models [27]. In the EM algorithm, the Kalman filter is employed to estimate state variables in the E step and the maximum likelihood method is used to estimate unknown parameters in the M step.

We propose a novel three-step procedure, the Expectation-Regularization-Maximization (ERM) algorithm, to estimate the high-dimensional sparse system matrix *A*, and other parameters, as well as the state variables for linear SSMs. The procedure is outlined as follows:

- E Step: the conditional expectation of the likelihood (4) is calculated by
  $$\begin{array}{c} G\left(\theta |\theta^{\left(r-1\right)}\right)=E_{X|Y,\theta^{\left(r-1\right)}}\left(\log P\left(\theta \right)\right), \end{array}$$ (5)
  where *θ*^(*r*−1)^ are the estimated parameters at the (*r* − 1)th iteration. In addition, the state variables (*x*_*t*_) and their sufficient statistics (functions of *x*_*t*_) required for estimating unknown parameters in the R and M step, are also estimated through the Kalman filter and smoother at this step.
- R Step: the L1 regularization, or the adaptive LASSO method, is employed to obtain the estimate of the sparse system matrix *A* denoted by *A*^(*r*)^.
- M Step: the MLE of other model parameters θ* = (*Q*, *R*, *μ*, *Σ*), denoted by θ*^(*r*)^, is obtained by maximizing the conditional expectation of likelihood
  $$\begin{array}{c} G\left(\theta^{*}|\theta^{\left(r-1\right)},A^{\left(r\right)}\right)=E_{X|Y,\theta^{\left(r-1\right)},A^{\left(r\right)}}\left(\log P\left(\theta \right)\right). \end{array}$$ (6)

For the standard SSM, the EM algorithm starts from the E Step for a given initial value of the system matrix *A* based on the prior knowledge of the dynamic system. However, for the high-dimensional linear SSM, it is not feasible to provide a good initial value for a high-dimensional sparse system matrix *A*. Thus, we recommend to start the proposed ERM algorithm from the R Step, which depends on an initial estimation of the state variable (*x*_*t*_) that can be obtained by a nonparametric local polynomial or spline smoother [28] instead of the Kalman filter. Thus, the proposed ERM algorithm should follow the order of R-M-E steps iteratively, or one R step, then E-R-M steps iteratively, until the log-likelihood estimates converge. The detailed implementation for each step is discussed in the following subsections.

#### E step: Kalman filtering and smoothing

The following sufficient statistics required for unknown parameter estimation in the R and M steps can be computed via the Kalman filter and smoother, *E*(*x*_*t*_|*y*_1_, …, *y*_*T*_), $E(x_{t}x_{t}^{\prime }|y_{1},\ldots ,y_{T})$ and $E(x_{t}x_{t-1}^{\prime }|y_{1},\ldots ,y_{T})$, which are denoted by $x_{t}^{T}$, ${\left(xx^{\prime }\right)}_{t}^{T}$ and ${\left(xx^{\prime }\right)}_{t,t-1}^{T}$; Var(*x*_*t*_|*y*_1_, …, *y*_*τ*_) by $V_{t}^{\tau }$ and $\text{Cov}(x_{t}x_{t-1}^{\prime }|y_{1},\ldots ,y_{\tau })$ by $V_{t,t-1}^{\tau }$, where *τ* is an arbitrary time point. The Kalman filter and smoother involve forward and backward recursions. In the forward recursions, estimation of the current states (filtering) and prediction of the next state are made based on the past measurements. For the backward recursions (smoothing), the past states are estimated given all the measurements up to the very last time point. Namely, the state variables are estimated by $x_{t}^{T}=E\left(x_{t}|y_{1},\ldots ,y_{T}\right)$. The general Kalman filtering and smoothing algorithms are given as follows.

- Forward recursions: Prediction and Filtering
  $$\begin{array}{ccc} x_{t}^{t-1} & = & Ax_{t-1}^{t-1},\\ V_{t}^{t-1} & = & AV_{t-1}^{t-1}A^{\prime }+Q \end{array}$$ (7)
  $$\begin{array}{ccc} K_{t} & = & V_{t}^{t-1}C^{\prime }{\left(V_{t}^{t-1}C^{\prime }+R\right)}^{-1},\\ x_{t}^{t} & = & x_{t}^{t-1}+K_{t}\left(y_{t}-Cx_{t}^{t-1}\right),\\ V_{t}^{t} & = & V_{t}^{t-1}-K_{t}CV_{t}^{t-1} \end{array}$$ (8)
  As we mentioned earlier, in the first iteration of the E step, *A* will be estimated by an initial R step. The initial state mean $x_{0}^{0}=\mu$ can be replaced by a small nonzero initial vector such as (0.1, 0.1, …, 0.1), and the variance matrix $V_{0}^{0}=\Sigma$ can be replaced by a small diagonal matrix, such as 10^−5^ × *I*_*p*×*p*_. *R*, *Q* can be initialized by two identity matrices—these two parameters will be updated in the M step.
- Backward recursions: Smoothing
  $$\begin{array}{ccc} J_{t-1} & = & V_{t-1}^{t-1}A^{\prime }{\left(V_{t}^{t-1}\right)}^{-1},\\ x_{t-1}^{T} & = & x_{t-1}^{t-1}+J_{t-1}\left(x_{t}^{T}-Ax_{t-1}^{t-1}\right)\\ V_{t-1}^{T} & = & V_{t-1}^{t-1}+J_{t-1}\left(V_{t}^{T}-V_{t}^{t-1}\right)J_{t-1}^{\prime }\\ V_{t-1,t-2}^{T} & = & V_{t-1}^{t-1}J_{t-2}^{\prime }+J_{t-1}\left(V_{t,t-1}^{T}-AV_{t-1}^{t-1}\right)J_{t-2}^{\prime } \end{array}$$ (9)
  where $V_{t-1,t-2}^{T}$ is initialized by $V_{T,T-1}^{T}=\left(I-K_{T}C\right)AV_{t-1}^{t-1}$.

Notice that the above algorithms require *p* × *p* matrix inverse calculations, which is computationally heavy and numerically unstable for a high dimensional system. To avoid such problem, Kojima *et al* [24] derived a recursive formula from the blockwise matrix inversion theorem. Matrices $V_{t}^{t}$ in Eq (8) and ${\left(V_{t}^{t-1}\right)}^{-1}$ in Eq (9) can be expressed alternatively as,

$$\begin{array}{ll} V_{t}^{t} & ={\left[C^{\prime }R^{-1}C+{\left(V_{t}^{t-1}\right)}^{-1}\right]}^{-1},\\ {\left(V_{t}^{t-1}\right)}^{-1} & =Q^{-1}-Q^{-1}A{\left[A^{\prime }Q^{-1}A+{\left(V_{t-1}^{t-1}\right)}^{-1}\right]}^{-1}A^{\prime }Q^{-1}. \end{array}$$

Note that the inverse of the matrix in brackets above are of the same form (*B*′Δ*B* + *D*^−1^)^−1^, where *D* is a symmetric *n* × *n* matrix, *B* is an arbitrary *n* × *n* matrix, and Δ is a diagonal matrix with diagonal elements *δ*_1_, …, *δ*_*n*_. Let *b*_*i*_ denote the *i*th row vector of *B*, *i* = 1, …, *n* and *D*_0_ = *D*, we can use the following recursive formula,

$$\begin{array}{c} D_{i+1}=D_{i}-\frac{1}{1/\delta_{i+1}+b_{i+1}^{\prime }D_{i}b_{i+1}}D_{i}b_{i+1}b_{i+1}^{\prime }D_{i+1}^{\prime } \end{array}$$

to calculate the inverse matrix (*B*′Δ*B* + *D*^−1^)^−1^, which is given by *D*_*n*_. Thus, $V_{t}^{t}$ and *J*_*t*−1_ in Eqs (8) and (9) can be calculated by the recursive formula without the inverse matrix calculations.

#### R step: L1 regularization using the adaptive LASSO

The implementation of the R Step in the proposed ERM algorithm is critical. The adaptive LASSO estimates can be obtained efficiently by using the computationally efficient LARS algorithm [29], which needs to be customized to the ERM algorithm. The extended BIC (eBIC) for large model selection proposed by [30] is employed to select the tuning parameters in the adaptive LASSO method. This model selection criterion is shown to be consistent under some mild conditions and also meets the needs of variable selection for larger model spaces [30].

LASSO [31] is a popular L1 regularization technique for performing estimation and variable selection simultaneously, which is consistent only under relatively restrictive mathematical assumptions. The adaptive LASSO proposed by [32], where adaptive weights are used for penalizing different coefficients in the L1 penalty, enjoys the desired oracle property under much weaker assumptions, namely, it performs as well as if the true underlying model were given in advance and produces asymptotically unbiased estimators for the nonzero parameters in linear regression models.

The adaptive LASSO estimates can be implemented by using the LARS algorithm [29]. In order to modify and apply the LARS algorithm for the R Step in the proposed ERM algorithm for linear SSMs, we need to use the vectorized matrix notations [33, 34], i.e., denote

$$\begin{array}{cc} X^{*}=(x_{2},x_{3},\ldots ,x_{T}), & X=\text{vec}(X^{*}), \end{array}$$ (10)

$$\begin{array}{cc} Z^{*}={\left(x_{1},x_{2},\ldots ,x_{t-1}\right)}^{\prime }, & Z=Z^{*}⊗I_{p\times p}, \end{array}$$ (11)

$$\begin{array}{cc} a^{*}=(a_{1},a_{2},\ldots ,a_{p}), & \alpha =\text{vec}(a^{*}), \end{array}$$ (12)

$$\begin{array}{cc} e^{*}=(w_{2},w_{3},\ldots w_{T}), & e=\text{vec}(e^{*}), \end{array}$$ (13)

where *vec* is the stack operator and ⊗ is the Kronecker product; *a*_*i*_ is the *i*th row vector of *A*; *α* is the vectorized *A* which is a (*p*^2^ × 1) vector; *e* is a (*p*(*T* − 1) × 1) vector that represents measurement errors.

### The matrix-based ERM algorithm

In the standard EM algorithm for linear SSMs, the estimate of *A*, the system matrix, can be obtained by maximizing the conditional expectation of the likelihood function (5), which is equivalent to minimizing

$$\begin{array}{c} G\left(\alpha \right)=E_{X,Z|Y,\theta^{\left(r-1\right)}}\left\{{\left(X-Z\alpha \right)}^{\prime }\left(X-Z\alpha \right)\right\}, \end{array}$$

in vectorized notations. This produces the MLE estimator of *A* as

$$\hat{A}=\left(\sum_{t=2}^{T}{\left(xx^{\prime }\right)}_{t,t-1}^{T}\right){\left(\sum_{t=1}^{T-1}{\left(xx^{\prime }\right)}_{t}^{T}\right)}^{-1}.$$ (14)

This estimator for the high-dimensional sparse matrix *A* is over parameterized and requires that $\left(\sum_{t=1}^{T-1}{\left(xx^{\prime }\right)}_{t}^{T}\right)$ is invertible. In this study, we propose to use an L1-regularized estimator of *A* that minimizes

$$\begin{array}{c} G\left(\alpha \right)=E_{X,Z|Y,\theta^{\left(r-1\right)}}\left\{{\left(X-Z\alpha \right)}^{\prime }\left(X-Z\alpha \right)\right\}+\lambda \sum_{j}{\hat{\text{w}}}_{j}\left|\alpha_{j}\right|, \end{array}$$ (15)

where λ is a tuning parameter. This is equivalent to applying the LASSO method to the restructured state equation in (2) in a matrix pseudo-regression form

$$\begin{array}{c} X=Z\alpha +e,\;e∼N(0,I_{\left(T-1)\times (T-1\right)}⊗Q)=N(0,\sigma_{Q}^{2}\cdot I_{(T-1)p\times (T-1)p}). \end{array}$$ (16)

We call this a pseudo-regression model since **X** and **Z** are state variables estimated from SSMs, instead of measured response variables and covariates in a standard regression model. Note that the elements of *e* are independent due to the assumption that *Q* is diagonal. If the state variables were directly observable without measurement error, model (16) is a standard first-order VAR model and one could simply apply the LASSO method to this VAR model [33, 34]. However, for the linear SSM, we need to use the sufficient statistics, $x_{t}^{T}$, ${\left(xx^{\prime }\right)}_{t}^{T}$, and ${\left(xx^{\prime }\right)}_{t,t-1}^{T}$ obtained from the E step to evaluate *E*(**Z**′**Z**) and *E*(**Z**′**X**) and the corresponding LARS algorithm needs to be modified accordingly. If *T* − 1 > *p*, the maximum likelihood estimator of *A* defined in Eq (14) is root-*n*-consistent and can be employed to determine the adaptive weights in the adaptive LASSO procedure as follows

$$\begin{array}{c} {\hat{\text{w}}}_{ij}={\left|{\hat{A}}_{ij}\right|}^{-1}. \end{array}$$ (17)

For “large *p*, small *n*” problems (*T* − 1 ⩽ *p*), we adopt a method developed for sparse high-dimensional regression in [35] by using the following marginal estimator of *A* instead of $\hat{A}$

$$\begin{array}{ll} {\hat{\text{w}}}_{ij}={\left|{\tilde{A}}_{ij}\right|}^{-1}, & \tilde{A}=\left(\sum_{t=2}^{T}{\left(xx^{\prime }\right)}_{t,t-1}^{T}\right)\;\text{diag}{\left(\sum_{t=1}^{T-1}{\left(xx^{\prime }\right)}_{t}^{T}\right)}^{-1}. \end{array}$$ (18)

Here $\text{diag}(\sum_{t=1}^{T-1}{\left(xx^{\prime }\right)}_{t}^{T})$ is a diagonal matrix of which the diagonal elements matches those of $\sum_{t=1}^{T-1}{\left(xx^{\prime }\right)}_{t}^{T}$. It was shown in [35] that under mild assumptions, $\tilde{A}$ is a zero-consistent estimator of *A*; using ${\hat{\text{w}}}_{ij}={\left|{\tilde{A}}_{ij}\right|}^{-1}$ as weight for adaptive LASSO can achieve oracle efficiency in variable selection.

Once *α* is estimated by adaptive LASSO, the system matrix ${\hat{A}}_{aL}$, can be easily reconstructed by “reshaping” the (*p*^2^ × 1) vector *α* into a (*p* × *p*) matrix. The elements in ${\hat{A}}_{aL}$ are shrunk toward zero as the L1 penalty parameter λ increases. Some elements are shrunk to exact zeros when λ is sufficiently large. Thus, it is important to determine the tuning parameter λ appropriately. We find that the use of standard AIC, BIC, cross-validation and other classical methods for determining λ tends to select a larger model for a high-dimensional model with sparse data, which may diminish the parsimonious property that we aim to preserve. The extended BIC [30] is recommended since it contains an extra penalty term with the consideration of different prior distributions over the model space.

#### M step: Maximization

The estimation of the remaining parameters is straightforward. The estimates of (*Q*, *R*, *μ*, Σ) can be obtained by maximizing the expected conditional likelihood (6) with given sufficient statistics from the E step and ${\hat{A}}_{aL}$ from the R step, *i.e.*,

$$\begin{array}{cc} \hat{\mu }=x_{1}^{T}, & \hat{\Sigma }={\left(xx^{\prime }\right)}_{1}^{T}-x_{1}^{T}x_{1}^{T^{\prime }}, \end{array}$$ (19)

$$\begin{array}{cc} \hat{R}=\frac{1}{T}\sum_{t=1}^{T}(y_{t}y_{t}^{\prime }-x_{t}^{T}y_{t}^{\prime }), & \hat{Q}=\frac{1}{T-1}\sum_{t=2}^{T}({\left(xx^{\prime }\right)}_{t}^{T}-{\hat{A}}_{aL}{\left(xx^{\prime }\right)}_{t,t-1}^{T}). \end{array}$$ (20)

### The row-based ERM algorithm

Note that in the above R Step implementation, *α* is a (*p*^2^ × 1) vector and **Z** is a (*p*^2^ × *p*^2^) matrix. Thus, it involves *p*^2^-dimensional matrix manipulations and computations. When *p* is large, it requires a massive amount of computer memory to carry out these computations, which may cause out-of-memory crashes of the proposed ERM algorithm. In this subsection, we propose a row-based approach for the R Step in the proposed ERM algorithm. Denote **X**_*i*_ as the *i*th row of X*, *a*_*i*_ as the *i*th row of *A*, and ***e***_*i*_ as the *i*th row of e*. The adaptive LASSO estimate of *A* can be obtained by equivalently applying the adaptive LASSO to a linear pseudo-regression model,

$$X_{i}=Z^{*}a_{i}+e_{i},$$ (21)

where

$$\begin{array}{c} Z^{*}=(\begin{array}{cccc} x_{11} & x_{12} & \ldots & x_{1p}\\ x_{21} & x_{22} & \cdots & x_{2p}\\ \vdots & \vdots & \ddots & \vdots \\ x_{(T-1)1} & x_{(T-1)2} & \cdots & x_{(T-1)p} \end{array}) \end{array}$$

is a (*T* − 1) × *p* matrix. Thus, the adaptive LASSO estimate of *a*_*i*_ (*i* = 1, …, *p*) is

$$\begin{array}{c} {\hat{a}}_{i_{aL}}=\arg\min_{a_{i}}E_{X,Z|Y,\theta^{\left(r-1\right)}}\left\{\right\|X_{i}-Z^{*}a_{i}{\|}^{2}+\lambda \sum_{ij}{\hat{\text{w}}}_{ij}|a_{ij}\left|\right\}, \end{array}$$ (22)

which only needs (*p* × *p*)-dimensional (instead of (*p*^2^ × *p*^2^)-dimensional) matrix manipulations. We use the same adaptive weights as in the matrix-based algorithm. Similarly, the sufficient statistics $x_{t}^{T}$, ${\left(xx^{\prime }\right)}_{t}^{T}$ and ${\left(xx^{\prime }\right)}_{t,t-1}^{T}$ from the E step are used in this step. Although we need to repeat the above LASSO procedure for each row *i* = 1, 2, …, *p*, the row-based ERM algorithm takes less time and less memory compared to the matrix-based algorithm. Below we describe our simulation studies done for comparisons of the performance between the row-based and matrix-based algorithms.

## Results

### Simulation studies

The proposed ERM algorithm for high-dimensional SSMs to construct dyamic MINs invovles estimation and regularization of a large number of parameters. We designed simulation studies to evaluate the methodology and the implementation procedure. We compared the row-based ERM algorithm to the matrix-based ERM algorithm, and we also evaluated the performance of the row-based ERM algorithm in more detail.

The row-based algorithm was proposed to overcome the computational limitation of the matrix-based algorithm as discussed in the previous section. We design the first simulation study for different number of dimensions *p* = 8, 20, 50, and 80. Thus, the total number of elements in the system matrix *A* is 64, 400, 2500, and 6400, respectively. The number of nonzero elements in *A* is assumed as 15, 35, 84, and 150 for the four cases, respectively. The nonzero elements were randomly generated from ±(0.4, 0.5, 0.6, 0.7, 0.8, 0.9). We also assume the variance parameters as *Q* = *I* and *R* = 0.1 × *I* for all the cases and the number of time points *T* = 60. As suggested, we applied and started the ERM algorithm from the R step to the 100 simulated data sets (*M* = 100).

To evaluate the performance of the proposed ERM algorithm for variable selection, we calculated the false positive rate (FP) and false negative rate (FN) of ${\hat{A}}_{aL}$ by

$$\begin{array}{ll} \text{FP}=\frac{\sum_{ij}1_{\left\{{\hat{a}}_{ij}\neq 0|a_{ij}=0\right\}}({\hat{a}}_{ij})}{\text{N}}, & \text{FN}=\frac{\sum_{ij}1_{\left\{{\hat{a}}_{ij}=0|a_{ij}\neq 0\right\}}1({\hat{a}}_{ij})}{\text{P}} \end{array},$$ (23)

where P is the number of nonzero elements and N is the number of zero elements in *A*, and $1_{\left\{\cdot \right\}}\left({\hat{a}}_{ij}\right)$ is an indicator function. In this simulation experiment, we fixed *Q* and *R* as their true values in order to have a fair comparison for the two algorithms. We report the average FP and FN over *M* = 100 simulation runs in Table 1.

**Table 1:** Simulation results: Comparisons of variable selection performance between the row-based and matrix-based ERM algorithms.

*p*	*p*^2^	% nonzero	algorithm	FP	FN
8	64	23.44	row	0.0459	0.0600
			matrix	0.0255	0.1073
20	400	8.75	row	0.0108	0.0531
			matrix	0.0095	0.0714
50	2500	3.36	row	0.0053	0.1458
			matrix	0.0039	0.1760
80	6400	2.34	row	0.0060	0.2306
			matrix	*N*/*A*	*N*/*A*

Table 1 shows that both row-based and matrix-based algorithms produce reasonable results. The matrix-based algorithm tends to yield a smaller false positive rate, but a larger false negative rate compared to that of the row-based algorithm. The false positive rate of the row-based algorithm is controlled very well although it is slightly larger than that of the matrix-based algorithm. The false negative rates for both algorithms are always higher (much higher for some cases) than the false positive rates. More importantly, a regular desktop machine running MATLAB ran out of memory estimating the 80×80 *A* matrix when the matrix-based algorithm is used, whereas the row-based algorithm can still perform reasonably well. Thus, we suggest using the row-base ERM algorithm for practical applications due to its efficiency and capability to handle high-dimensional matrices.

In the third simulation experiment, we evaluate the performance of the proposed row-based ERM algorithm for different sample sizes and the effect of system noise *Q*. Since the system noise *Q* and measurement noise *R* cannot be identified simultaneously based on the single sequence data without replication, we decided to fix *R* in this simulation to avoid the identifiability problem. The true system matrix for the SSM in this simulation experiment is a 41×41 system matrix *A*. We generated equally-spaced temporal data with the number of time points *T* = 20, 50 and 100 for each bacterial OTU. We expect to see the improved estimation with increased sample sizes. For the variance of the system noise *Q*, we compared the two cases: fixed as the true value or estimated from the data. Total of *M* = 100 data sets were simulated for each scenario. The simulation results are reported in Table 2.

**Table 2:** Evaluation of the row-based ERM algorithm for variable selection with respect to number of time points T and Q estimation. *p* = 41.

T	Q	R	FP	FN
20	fixed as true	fixed as true	0.0167	0.3111
	estimated	fixed as true	0.0192	0.3479
50	fixed as true	fixed as true	0.0151	0.1617
	estimated	fixed as true	0.0150	0.1803
100	fixed as true	fixed as true	0.0189	0.0915
	estimated	fixed as true	0.0180	0.0984

Table 2 shows that, as the number of time points *T* increased from 20 to 100, the false negative rate significantly decreased from 0.31 or 0.35 to 0.092 or 0.098 for fixed or estimated *Q*, while the false positive rate roughly was stabilized at 0.015 to 0.019. Overall, for all three choices of *T*, fixing the system noise *Q* as the true value rather than estimating *Q* only reduced the false negative rate slightly, and it did not have much effect on the false positive rate. The adaptive LASSO procedure equipped with the eBIC method for tuning parameter selection seems to have enough power to identify important state variables in the SSMs. With more data, it becomes less conservative and selects more variables with a higher accuracy as expected. In summary, the proposed row-based ERM algorithm produces promising results for SSM variable selection and is computationally efficient. The false positive rate is not affected by the system noise or measurement noise, but the false negative rate can be improved if the true system noise or measurement noise can be accurately determined.

### Applications to microbiota data

In one recent longitudinal microbiome study, mid-vaginal swabs from 32 nonpregnant, reproductive-age women were obtained twice weekly, over a period of 16 weeks [4] during which each subject’s sexual and menstrual activity was also tracked. For each of the samples, variable regions of 16S ribosomal RNA gene were sequenced, yielding abundance estimates of bacterial species/genera (OTUs). Although sexual activity and menses showed impact on bacterial diversity, no clear relationships between environment and abundance of specific types or species of bacteria were identified, suggesting a likely causal role of the inter-microbial interactions. Several studies have characterized the normal bacterial communities in human vagina [4, 36–38]. Normal vaginal flora can be clustered into five to six groups based on their composition, most of which are dominated by lactic acid bacteria, and remaining few by anaerobic bacteria. Many members of the vaginal flora are highly specialized for the vaginal eco-niche indicating that the vaginal microbiome generates meaningful MINs [39]. We employ the proposed models to investigate the dynamic interactions among bacteria in this study, and infer the MINs for each subject.

Based on our simulation studies, we decided to use the extended BIC [30] to preserve the parsimonious property, and employ the row-based ERM algorithm for adaptive LASSO. As a matter of fact, we also tried the matrix-based ERM algorithm with extended BIC for this particular data set. Whilee the matrix-based algorithm yielded reasonable results in simulation studies, it shrank almost all the coefficients in the system matrix to zero for both subjects used in our study, which resulted in very poor fits. In contrast, row-based algorithm produced reasonable results in terms of fitting, and can be used effectively to infer MINs.

The magnitude of abundance varies widely for different bacteria. For example, for Subject 6, relative abundance of *Atopobium* (averaged over 27 days) is 1,542 times greater than that of *L. crispatus*, which is a known important beneficial species in vagina [40, 41]. Without proper standardization, a uniform L1 penalty is much more likely to set the edges related to less abundant OTUs to zero, and results in a simplistic network dominated by a few most abundant OTUs In order to make the results comparable, the measurements are standardized before the row-based ERM method is applied, *i.e*., we define $Y_{ij}=\frac{{\tilde{Y}}_{ij}-{\bar{Y}}_{i}}{\text{sd}({\tilde{Y}}_{i\cdot }))}$, where ${\tilde{Y}}_{ij}$ is the *j*th raw measurement for the *i*th subject, ${\bar{Y}}_{i}$ is the mean of ${\tilde{Y}}_{ij}$, and $\text{sd}({\tilde{Y}}_{i\cdot })$ is the standard deviation of ${\tilde{Y}}_{i\cdot }$.

Ideally, time course microbiome data with technical or biological replicates at each time point for each subject would allow investigators to use statistical methods to provide more reliable MIN structure identification and estimation. Microbiome studies often lack such replicates. Our method is still useful in this scenario, although the variance of the system noise and the measurement noise may not be estimable and identified simultaneously for such datasets.

### Inference of microbial interaction networks (MINs)

We applied the ERM method to infer MINs from the data published by [4]. Although we examined the available data for all the 32 subjects from this vaginal microbiome study, due to space limitation, here we report the results from two subjects: subject 15 and subject 6, because these two subjects cover very different vaginal microbiome profiles. For any given subject, the abundance measurements for most bacteria are zero. For consistency and simplicity, we selected only those bacteria for which at least 30% of the abundance measurements across the 16 weeks of study are nonzero. Based on this criterion, Subject 6 had 34 and Subject 15 had 12 bacterial OTUs which were identified for modeling.

**Fig 1:**
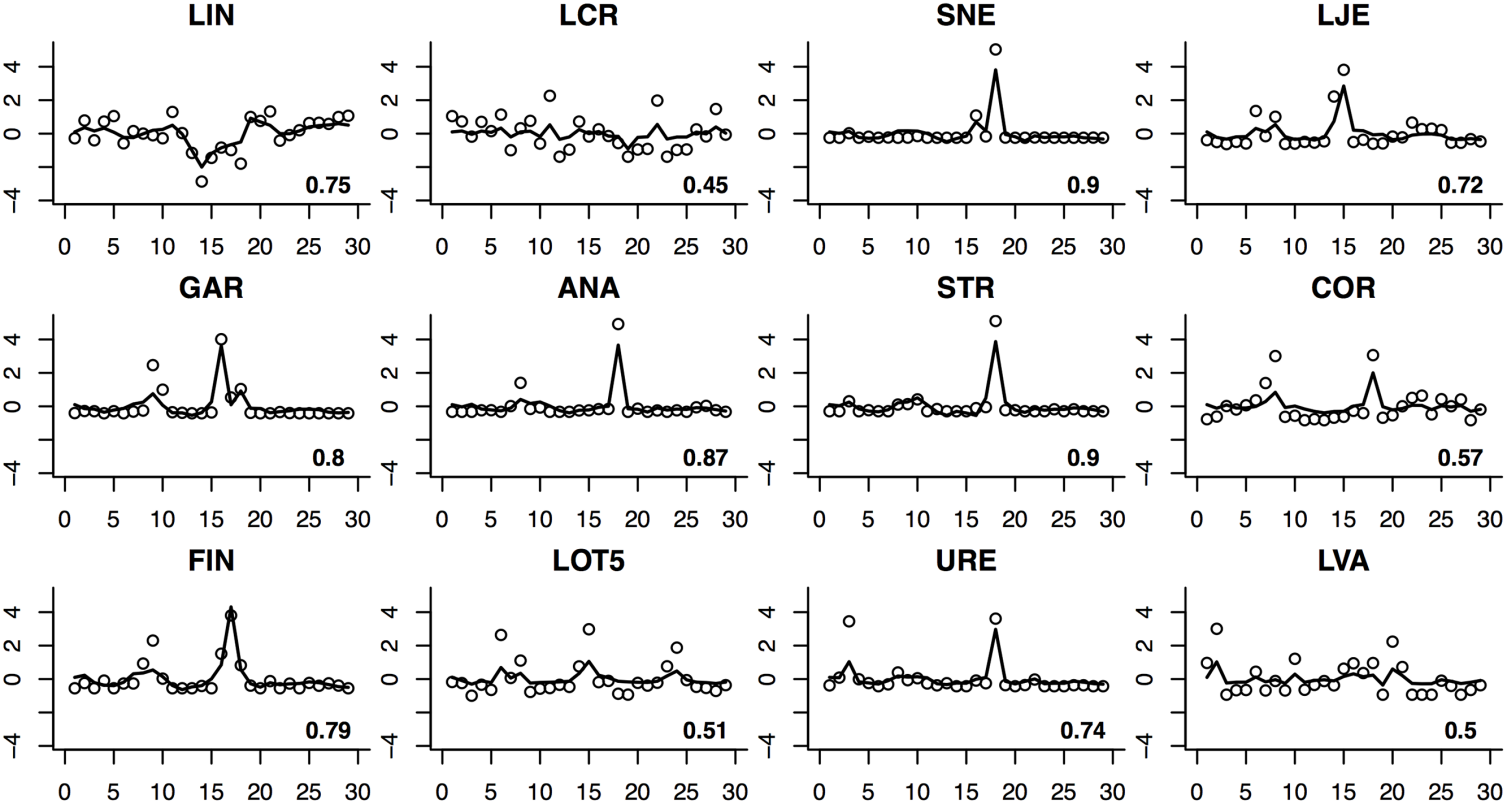
One-step-ahead prediction for subject 15. Each cell represents prediction for a different OTU. Solid lines depict the predicted values whereas circles indicate standardized temporal abundances of OTUs. Abbreviations for Operational Taxonomic Units: LIN *Lactobacillus iners*; LCR *Lactobacillus crispatus*; SNE *Sneathia sp.*; LJE *Lactobacillus jensenii*; GAR *Gardnerella sp.*; ANA *Anaerococcus sp.*; STR *Streptococcus sp.*; COR *Corynebacterium sp.*; FIN *Finegoldia sp.*; LOT5 *Lactobacillus otu5*; URE *Ureaplasma sp.*; LVA *Lactobacillus vaginalis*.

**Fig 2:**
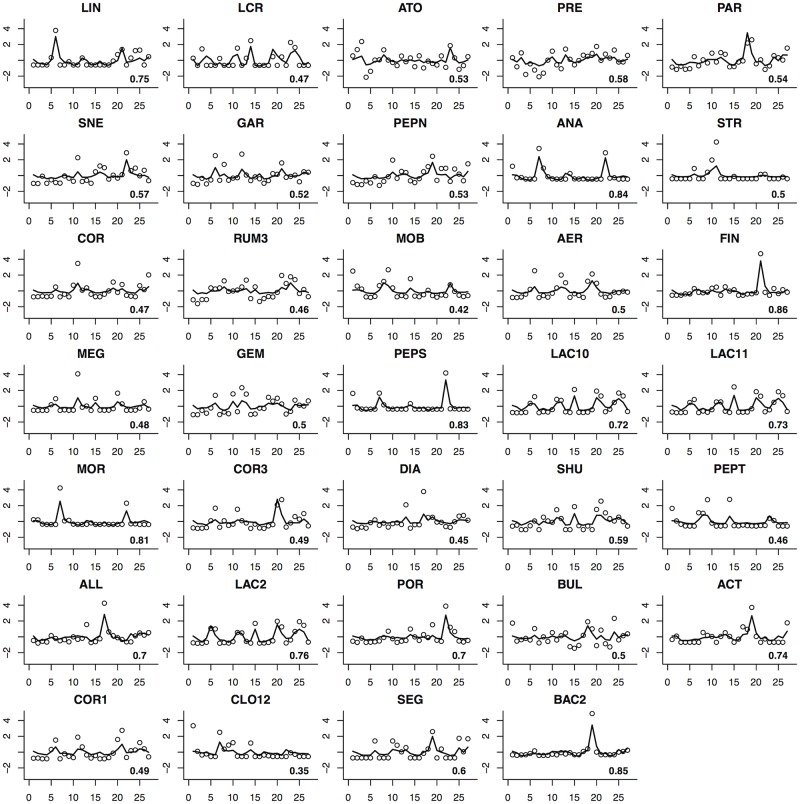
One-step-ahead prediction for subject 6. Each cell represents prediction for a different OTU. Solid lines depict the predicted values whereas circles indicate standardized temporal abundances of OTUs. Abbreviations for Operational Taxonomic Units: LIN *Lactobacillus iners*; LCR *Lactobacillus crispatus*; ATO *Atopobium sp.*; PRE *Prevotella sp.*; PAR *Parvimonas sp.*; SNE *Sneathia sp.*; GAR *Gardnerella sp.*; PEPN *Peptoniphilus sp.*; ANA *Anaerococcus sp.*; STR *Streptococcus sp.*; COR *Corynebacterium sp.*; RUM3 *Ruminococcaceae 3*; MOB *Mobiluncus sp.*; AER *Aerococcus sp.*; FIN *Finegoldia sp.*; MEG *Megasphaera sp.*; GEM *Gemella sp.*; PEPS *Peptostreptococcus sp.*; LAC2 *Lachnospiraceae 10*; LAC11 *Lachnospiraceae 11*; MOR *Moryella sp.*; COR3 *Coriobacteriaceae 3*; DIA *Dialister sp.*; SHU *Shuttleworthia sp.*; PEPT *Peptococcus sp.*; ALL *Allisonella sp.*; LAC10 *Lachnospiraceae 2*; POR *Porphyromonas sp.*; BUL *Bulleidia sp.*; ACT *Actinomyces sp.*; COR1 *Coriobacteriaceae 1*; CLO12 *Clostridiales 12*; SEG *Segniliparus sp.*; BAC2 *Bacteroidales 2*.

Figs 1 and 2 present the one-step-ahead prediction for these two subjects, based on the estimated SSM model. We also calculated the coefficients of determination (*R*^2^) for all the bacteria involved in the MINs for each subject (in lower right corner of each plot in Figs 1 and 2). The predictions look reasonable and there is no apparent evidence of overfitting, which is a common pitfall in high-dimensional data analysis.

**Fig 3:**
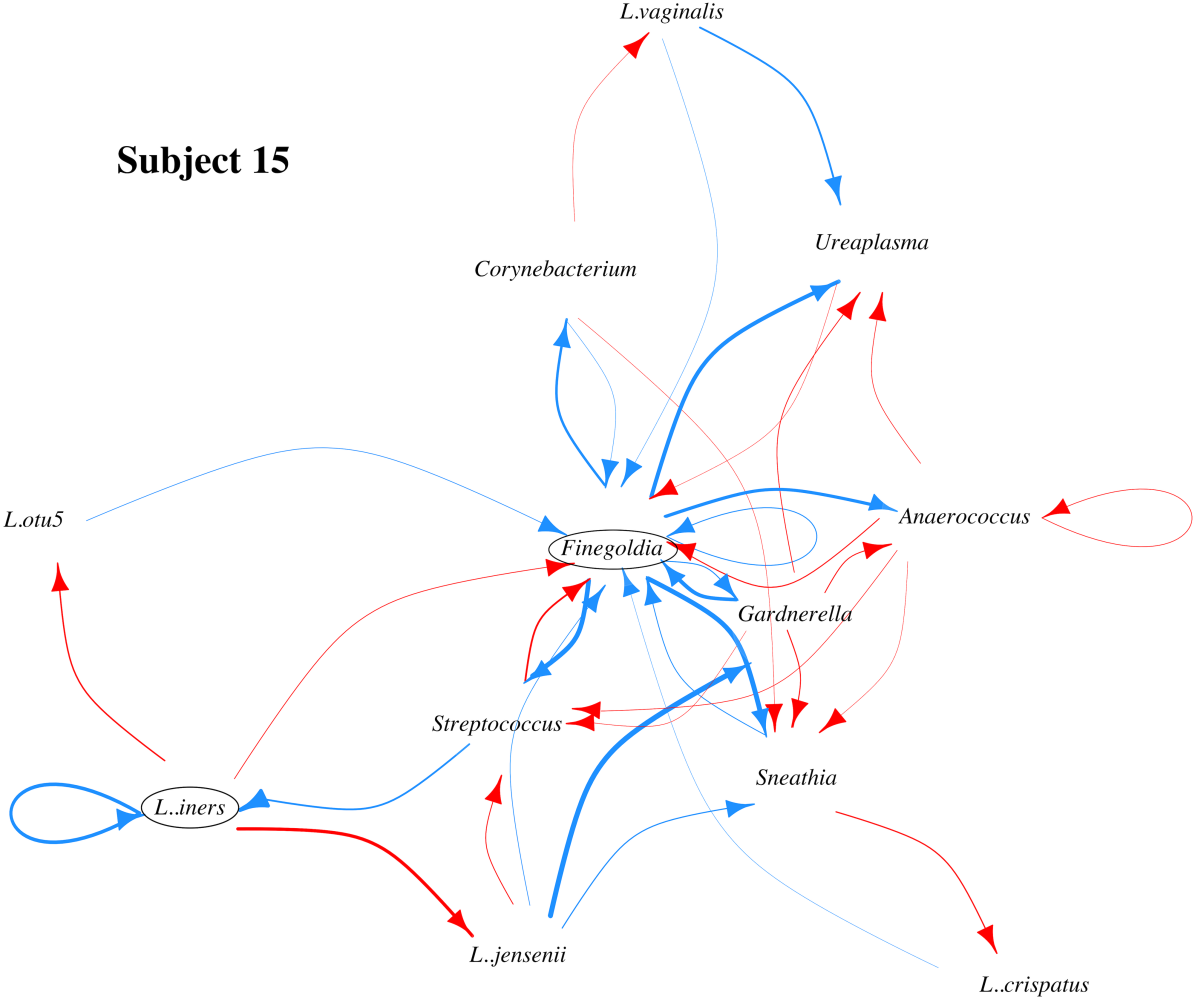
Microbial interaction network (MIN) for subject 15. Blue and red arrows indicate directed positive and negative effects respectively. Arrow width indicates effect magnitude. Circles highlight bacterial species that impact multiple other species in the MIN and whose critical role in the MIN has either experimental support in literature (*L. iners*) or has never been recognized before (*Finegoldia sp.*).

**Fig 4:**
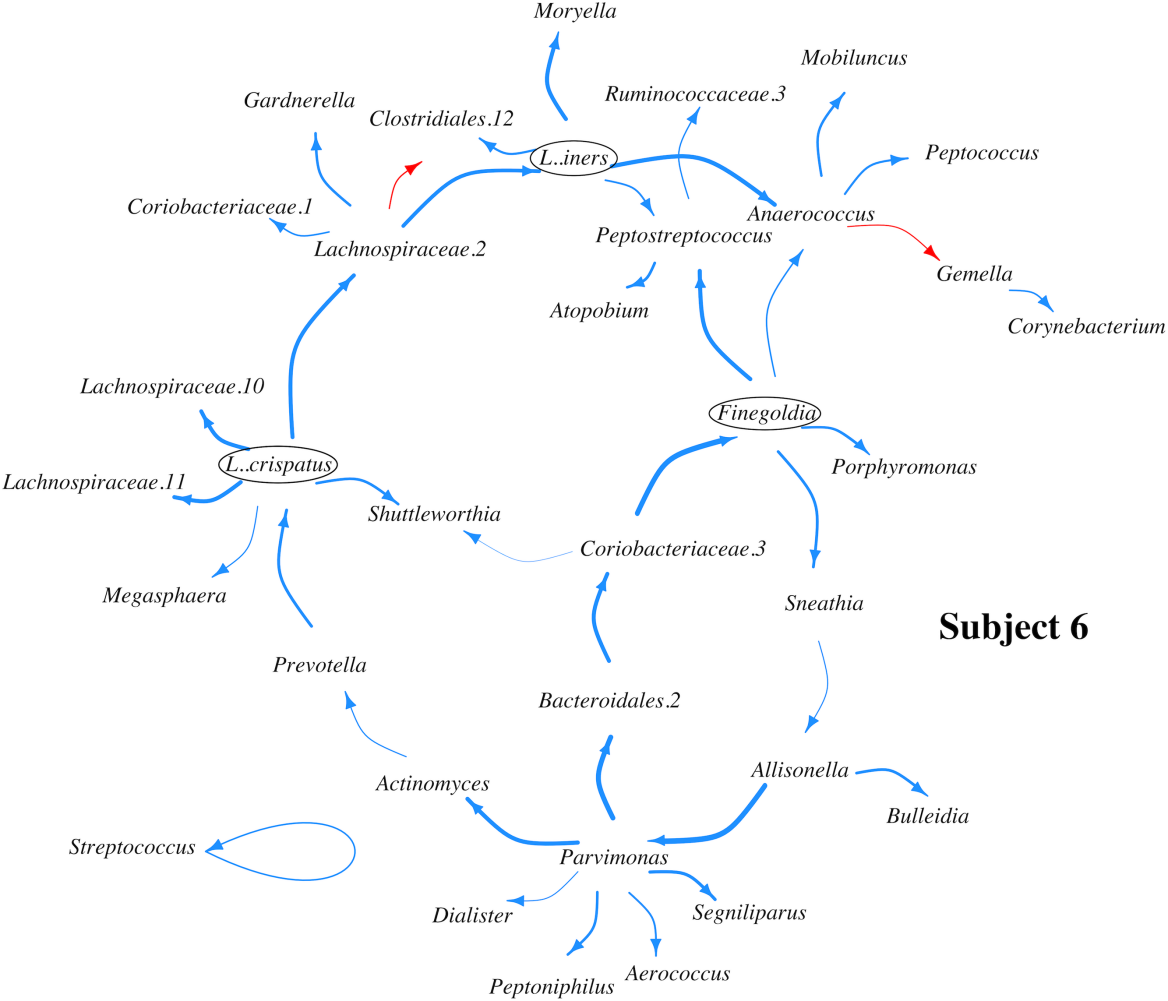
Microbial interaction network (MIN) for subject 6. Blue and red arrows indicate directed positive and negative effects respectively. Arrow width indicates effect magnitude. Circles highlight bacterial species that impact multiple other species in the MIN and whose critical role in the MIN has either experimental support in literature (*L. iners*) or has never been recognized before (*Finegoldia sp.* and *L. crispatus*).

The inferred MIN for the two subjects, are reported in Tables 3 and 4, as well as in Figs 3 and 4.

**Table 3:** Interactions among bacteria for subject 15. See legend for Fig 1 for OTU abbreviations.

Bacteria	Positive Effects	Negative Effects
LIN	LIN	LJE, FIN, LOT5
LCR	FIN	
SNE	FIN	LCR
LJE	SNE, GAR, FIN	STR
GAR	FIN	SNE, ANA, STR, URE
ANA		SNE, ANA, STR, FIN, URE
STR	LIN	FIN
COR	FIN	SNE, LVA
FIN	SNE, GAR, ANA, STR, COR, FIN, URE	
LOT5	FIN	
URE		FIN
LVA	FIN, URE	

**Table 4:** Interactions among bacteria for subject 6. See legend for Fig 2 for OTU abbreviations.

Bacteria	Positive Effects	Negative Effects
LIN	ANA, PEPS, MOR, CLO12	
LCR	MEG, LAC10, LAC11, SHU, LAC2	
PRE	LCR	
PAR	PEPN, AER, DIA, ACT, SEG, BAC2	
SNE	ALL	
ANA	MOB, PEPT	GEM
STR	STR	
FIN	SNE, ANA, PEPS, POR	
GEM	COR	
PEPS	ATO, RUM3	
COR3	FIN, SHU	
ALL	PAR, BUL	
LAC2	LIN, GAR, COR1	CLO12
ACT	PRE	
BAC2	COR3	

The number of edges from each bacterial OTU ranges from one to seven. There are a total of 38 (25.7%) nonzero elements in the reconstructed system matrix A for subject 15, and 37 (3.3%) nonzero elements in the system matrix A for subject 6, both of which are sparse matrices and consistent with the sparsity property of MINs. As reported in [4], we note that the composition of vaginal flora of these two subjects differs significantly: subject 15 microbiome is dominated by *Lactobacillus iners*, and that of subject 6 is dominated by many anaerobic bacteria. Gajer et al. [4] classified vaginal microbiota of their subjects into five community types based on their bacterial compositions. Community types I to III were found to be dominated by *L. crispatus*, *Lactobacillus gasseri* and *L. iners*, respectively. Community type IV-A were dominated by moderate proportions of either of the many Lactobacillus species typically found in vagina. Community type IV-B tends to be dominated by a collection of diverse bacteria, all of which are present in large abundance. According to this classification, the vaginal microbiome of Subject 15 is dominated by community state type III, and that Subject 6 is dominated by community type IVB. Parts of the MINs that we obtained from these two subjects are in agreement with some previous experimental investigations into interactions among vaginal bacteria, and some inferred relationships are novel and unexpected.

#### Subject 15

We find that in the vaginal flora of Subject 15, *L. iners* is not just the predominant member, but also actively inhibits the proliferation of other lactobacilli, most prominently, that of *Lactobacllus jensenii* (Fig 3). *L. iners* reportedly shares reciprocal interference with a different lactobacillus (*L. gasseri*), but our results indicate that this interference may be phylogenetically more widespread [42]. We also find that *L. jensenii* actively aids the proliferation of *Gardnerella Sp.*, which has been implicated in bacterial vaginosis.

#### Subject 6

First we infer that *Finegoldia sp.*, an anaerobic bacterium belonging to class Clostridia directs the growth of multiple anaerobic bacteria that are more abundant than itself. The synergistic interactions of *Finegoldia sp.* with *Sneathia sp.* and with *Anarococcus sp.* were also identified in MIN of Subject 15. In fact *Finegoldia sp.* seems to occupy an influential position in both the MINs, in spite of its low abundance in both the subjects. This is one of the classical hallmarks of a “keystone” species in an ecosystem [43], and *Finegoldia spp.* has never been noted as one before. Second, Gajer et al. [4] identified that communities dominated by *L. iners* often appear to shift to one dominated by *Atopobium*, *Prevotella*, *Parvimonas*, *Sneathia*, *Gardnerella*, or *Mobiluncus*. We find that even in a microbial community in which *L. iners* is a minor component, it promotes the growth of some of these bacteria directly as well as indirectly, as in the inferred MIN of Subject 6. Third, *L. crispatus*, an aerobic, hydrogen peroxide producing bacterium that is normally associated with establishing normal and aerobic microflora, is inferred to be promoting growth of a number of facultatively anaerobic *Lachnospiraceae* species, which is a novel and unexpected outcome of the inferred MIN, and may warrant future experimental validations.

## Discussion

Dimensionality has always been a difficulty in identifying a complex microbial interaction network (MIN) due to the large number of bacteria observed in human microbiome. In this article, we proposed a new Expectation-Regularization-Maximization (ERM) algorithm for a high-dimensional linear state space model (SSM) to construct dynamic MINs from time course microbiome data. The Kalman filters in conjunction with the adaptive LASSO variable selection technique enable promising parameter estimation for the sparse high-dimensional microbiome interaction matrix. The implementation of the adaptive LASSO for the SSMs in the R step is shown to be straightforward and clean. To overcome the difficulty for large matrix manipulations for the high-dimensional SSMs, a row-based ERM algorithm was proposed and evaluated against the standard matrix-based algorithm. Our simulation and real data analysis results show that the row-based algorithm performs quite well. The proposed method can be easily adapted to accommodate data with longitudinal replicates. We successfully applied the method to time course vaginal microbiome data to construct dynamic MINs.

Similar VAR models with L1 regularization have been proposed for dynamic networks [33, 34]. However, the VAR model does not take measurement error into consideration. The SSMs equipped with the proposed ERM algorithm allow us to consider the system error and measurement error separately while constructing the dynamic network. Through the ERM algorithm, the variance of both errors can be estimated if the longitudinal data or biological replicates are available. A similar SSM representation with L1 regularization for dynamic gene regulatory network construction was proposed in [24]. However, an *ad hoc* method instead of the LARS algorithm was used in LASSO estimation which may reduce its performance. Furthermore, the program, or its implementation details, were not provided. In this paper, we fill the gap to clarify the methodological issues and provide a complete and simple implementation procedure. We also discussed how to initiate the proposed ERM algorithm from a practical perspective. From the computational perspective, we validated that the row-based ERM algorithm performs well for data analysis and recommended for practical use. Based on the proposed SSM and ERM algorithm, we establish the MIN for vaginal microbiome of women and some encouraging findings have been revealed.

We believe that our work is just the first step to reconstruct MIN using SSM model. Below we list a few weaknesses of our current method and possible directions for future work.

The state-space model considers time as discrete steps instead of a continuous variable as used in many alternative network models. As such, it is more resilient to temporal discontinuities and can be used to fit granular data and data with sharp jumps (discontinuities), which is demonstrated in Figs 1 and 2. That being said, we must point out that both system and measurement noises (*w*_*t*_ and *v*_*t*_ in Eq (1)) are modeled as Gaussian distributions in our current model, therefore we strongly suggest that our method should only be applied to modeling the interactions between key OTUs with relatively low sparsity. It will be very interesting to incorporate a proper discrete distribution such as the negative binomial distribution into the SSM model in the future. Secondly, we have only considered estimating MINs for each individual subjects. It is more meaningful to construct the common MIN for a population. This calls for more advanced SSM methods and data with more replications and better quality. Although convergence was not a big issue for the proposed algorithm in both simulations and real data analyses, we acknowledge that the addition of the R step may change the theoretical properties of the EM algorithm, which warrants further investigations. Exogenous variables such as gender, age, race *et al* can also be included in a more complex model. Nonlinear SSM models have been widely studied in recent years. The extension to nonlinear SSM models in conjunction with variable selection techniques also deserve further investigation. It would be interesting to compare the proposed model and method to alternative models and methods for dynamic network construction. Finally, we must point out that due to the large number of unknown parameters (*k*^2^ edges) in a high-dimensional network, inevitably there will be a certain number of false positives despite the best practice in model selection. It is therefore critical to conduct subsequent confirmatory experiments to validate the predicted interactions. The power of high-dimensional network models, such as the one proposed in this study, is that they help experimentalists generate high-quality hypotheses and select the most promising experiments to perform.
